# Assessing the feasibility of a bowel management program for patients with neurogenic bowel function after spinal cord injury

**DOI:** 10.3389/fmed.2025.1499184

**Published:** 2025-02-05

**Authors:** Jiayi Feng, Xingyi Mu, Lei Xu, Tongxia Xia

**Affiliations:** ^1^Nursing Department, Affiliated Hospital of Zunyi Medical University, Zunyi, Guizhou, China; ^2^Nursing College, Zunyi Medical University, Zunyi, Guizhou, China; ^3^Guiyang Public Health Clinical Center, Guiyang, Guizhou, China

**Keywords:** spinal cord injury, neurogenic bowel dysfunction, bowel management, assessing, feasibility

## Abstract

The aim of this study was to assess the feasibility of a bowel management program for patients with neurogenic bowel function after spinal cord injury. The program is based on evidence-based nursing, expert meeting and pre-experiment construction, the construction process is standardized and scientific, and the content is comprehensive, mainly includes 4 dimensions of bowel assessment, bowel intervention, assessment indices and discharge follow-up, which were carried out at the time of admission (T1), discharge (T2) and 1 month after discharge (T3) of patients in the experimental group, while the control group used routine orthopedic bowel management, and bowel function indices, quality of life and laboratory tests were used as outcome indices, and differences in the observed indices of patients in the two groups were compared to validate the effect of the program. Compared with the control group, the incidence of bloating, constipation and fecal incontinence was significantly reduced in the experimental group, while the frequency of defecation scores, fecal character scores, Neurogenic Bowel Dysfunction scores, laboratory test results and quality of life were also effectively improved. The results also highlight the need for a large, multi-center, long-term follow-up study to validate the efficacy of this protocol to improve the feasibility of bowel management protocols for patients with neurogenic bowel function after spinal cord injury. This study provides a reference base for further exploration of bowel management in patients with neurogenic bowel function after spinal cord injury and is worthy of promotion and application in clinical practice.

## 1 Introduction

Spinal cord injury (SCI) refers to motor, sensory, and reflex dysfunction caused by damage to the spinal cord’s structure or function (1, 2). In recent years, the incidence of SCI has risen due to an aging population, increased car accidents, and work-related injuries (3, 4). The available evidence suggests that the incidence of SCI in China ranges from 23.7 to 60.6 per million people per year (3), which is generally consistent with the global incidence of 10.4 to 80.0 per million people per year and the North American incidence of 20.7 to 83.0 per million people per year (5, 6). However, China’s large population has resulted in nearly 230,000 new cases each year, accounting for about 50% of new cases worldwide (4, 6, 7). Although spinal cord injury is considered a rare disease globally, the large number of cases in China results in a much higher challenge for the Chinese healthcare system than in other countries. These factors highlight the importance of localized epidemiological studies to address SCI’s unique burden in China. SCI primarily affects men, with the highest incidence between ages 20–29. In women, the highest risk occurs between ages 15–19 and over 60 (6). Overall, the incidence in men is twice that in women. SCI not only causes physical and psychological trauma but also imposes a significant economic burden on patients, their families, and society (8). The lifetime treatment cost for SCI patients can range from $1.1 million to $4.7 million, with each hospitalization costing between 20,000 and 50,000 yuan, and in some cases up to 300,000 yuan (9). Rehabilitation costs add further financial strain, averaging 20,000 yuan per session (10). The higher incidence and high cost of treatment place a serious burden on patients and their families.

Mortality in SCI patients is highest within the first year after injury and increases with the severity of the injury (11). Although current treatments can reduce mortality rates, SCI patients remain vulnerable to various complications, including “Neurogenic Bowel Dysfunction (‘NBD’),” deep vein thrombosis, and autonomic dysreflexia. Among these, bowel dysfunction is a significant issue. Studies show that the readmission rate for SCI patients within one year of discharge is 55% (12), with bowel problems accounting for over 30% of these admissions (13, 14).

NBD is a common and serious complication in SCI patients, characterized by a loss of sensory and/or motor control, leading to defecation issues (15). The Enteric Nervous System (ENS) regulates bowel motility, and NBD disrupts this regulation, causing severe bowel damage (12). Practically all spinal cord lesions, particularly complete injuries, can lead to NBD. While injuries to the sacral segment or cauda equina result in loss of reflexes and anal sphincter tone, causing symptoms such as abdominal distension, stool retention, and constipation, injuries above the sacral segment often cause fecal incontinence due to a lack of contractile control of the external anal sphincter. The prevalence of NBD in SCI patients is as high as 54%, with around 80% experiencing constipation, 43% experiencing abdominal distension, 38% experiencing abdominal pain, and over 5% experiencing fecal incontinence (12, 15–17).

Research indicates that most SCI patients suffer from mild to moderate bowel dysfunction, which can worsen over time if not properly managed (18). In severe cases, NBD can become life-threatening. Current studies on managing NBD focus on individual interventions or rehabilitation care, lacking a comprehensive approach. The “pyramid” principle of treatment is often used, involving diet and water plans, bowel training, medication, enemas, and surgical treatments. However, these methods may not adequately assess the patient’s bowel function and overall health, leading to ineffective bowel management (19, 20). Effective bowel function restoration is critical not only for treating NBD but also for improving patients’ quality of life (21). Research suggests that while some NBD patients can manage their condition with conservative treatments, the majority require medication, and many need assistance from others for bowel care (19). Inadequate management exacerbates symptoms, contributing to increased anxiety and depression in patients (22).

Given these challenges, our study aims to develop a comprehensive NBD management program for SCI patients. By focusing on scientific and holistic approaches, we seek to reduce complications, enhance patients’ quality of life, and improve their overall well-being.

## 2 Materials and methods

### 2.1 Study design

This study employed an quasi-experiment research method to investigate NBD in patients with SCI. The subjects were patients admitted to the Department of Spine Surgery at the Affiliated Hospital of Zunyi Medical University, between April 2022 and November 2023.

### 2.2 Sample size calculation

Based on a clinical study by Wei Xiaomei (23), the sample size was calculated using bloating incidence as a reference. In the experimental group, the incidence of bloating after intervention was P1 = 0.108, while in the control group, it was P2 = 0.353. With an *α* = 0.05, and a power (1 - *β*) = 0.80, and a 1:1 ratio between the control and experimental groups. PASS 15.0 software was used for the sample size calculation. The result showed that 43 patients were required for each group. Accounting for a 10% dropout rate, the final sample size was set at 48 patients per group, totaling 96 participants.

### 2.3 Inclusion and exclusion criteria

#### 2.3.1 Inclusion criteria

(1) Diagnosed with SCI, confirmed by CT or MRI, according to the American Spinal Cord Injury Association’s International Standard for Neurological Classification of SCI; both complete and incomplete SCI patients were included to account for potential differences in neuro-dysfunctional and co-morbid aspects; (2) Diagnosis of neurogenic bowel dysfunction as per Rome III criteria and the International Gastroenterology Organization guidelines; (3) Age 18 years or older; (4) No mental or cognitive impairments, with the ability to communicate clearly; (5) Voluntary participation with signed informed consent.

#### 2.3.2 Exclusion criteria

(1) Patients with other chronic bowel diseases; (2) Patients with significant heart, lung, liver, or kidney conditions; (3) Patients with anal fissures, perianal abscesses, or hemorrhoids; (4) Patients who develop severe complications during treatment; (5) Patients receiving treatments that could affect the study; (6) Those unwilling to cooperate or with cognitive disorders.

#### 2.3.3 Loss criteria

(1) Interruption of the study or voluntary withdrawal; (2) Loss of contact during follow-up.

### 2.4 Patient recruitment and grouping

The study recruited 96 inpatients with NBD post-SCI from the Department of Spine Surgery at the Affiliated Hospital of Zunyi Medical University, based on the inclusion criteria. A non-simultaneous control design was used, with patients from April 2022 to January 2023 forming the control group, and those from February 2023 to November 2023 forming the experimental group.

### 2.5 Intervention method

#### 2.5.1 Control group

Patients received standard orthopedic bowel care. This included health education for patients and their families, dietary advice to increase the intake of fresh vegetables, fruits, and high-fiber foods, and guidance to avoid gas-producing foods like milk and eggs. Medication was provided for patients who had not defecated for three days or who experienced constipation or other bowel issues.

#### 2.5.2 Experimental group

Implementation of bowel management program is detailed in Annex 1. First, a multidisciplinary intervention team was formed, including a senior nurse, three deputy senior nurses, an orthopedic surgeon, a rehabilitation nurse, a dietitian, a counselor, and three postgraduate nurses. The orthopedic surgeon primarily provided medical assessments related to spinal cord injuries and advised on any surgical or medical interventions necessary for bowel dysfunction. The rehabilitation nurse contributed expertise in physical and rehabilitation medicine, ensuring integration of physical therapy techniques into the bowel management program. The senior nurse oversaw project progress and quality control, while the deputy senior nurses coordinated and supervised the unit’s work. The dietitian and counselor supported the project’s implementation and provided professional guidance. The postgraduate nurses were responsible for carrying out the intervention and collecting and verifying data. Team leader members received unified training before intervention, including research content, research purpose and method, implementation process and precautions, and explained the content of bowel management program, including how to intervene, intervention time, intervention method and intervention content. In addition, in order to ensure that team members fully grasp the content and process of the program, irregular training is also conducted after the implementation of the program, so as to improve the reliability and effectiveness of the research.

### 2.6 Measuring indicators

#### 2.6.1 Primary outcome

##### 2.6.1.1 Constipation

Defined by ≥2 criteria from the Rome III diagnostic criteria: fewer than 3 bowel movements per week, lumpy or hard stools in ≤25% of bowel movements, a sensation of incomplete evacuation in ≤25% of movements, difficult defecation in ≤25% of movements, or a sensation of anal obstruction (24).

##### 2.6.1.2 Fecal incontinence

Unplanned or involuntary bowel movements occurring ≥1 time in the past month (25).

##### 2.6.1.3 Stool consistency

Assessed using the Bristol Stool Scale, with types 4–7 scored 0, type 3 scored 1, type 2 scored 2, and type 1 scored 3 (26).

##### 2.6.1.4 NBD score

Evaluated using a tool covering clinically relevant information about NBD in SCI patients, with scores ranging from 0–47. The NBD score consists of 10 items: frequency of bowel movements (0–6 points), time spent in each bowel movement (0–7 points), discomfort with bowel movements, headache, or sweating (0–2 points), regular use of medications to treat constipation (0–2 points) or regular use of drops to prevent constipation (0–2 points), rectal stimulation or fingers to promote bowel movements (0–8 points), frequency of fecal incontinence (0–13 points), medications to treat incontinence (0–4 points), gastrointestinal flatulence urinary incontinence (0–2 points), and perianal skin problems (0–3 points), incontinence (0 to 4 points), gastrointestinal flatulence incontinence (0 to 2 points), and perianal skin problems (0 to 3 points). The total NBD score ranges from 0 to 47 points, with higher scores suggesting more impairment of bowel function (27). Higher scores indicate more severe bowel symptoms.

#### 2.6.2 Secondary outcome

##### 2.6.2.1 Quality of life

Measured using the SF-12, a standardized tool with 12 items across 8 dimensions, including general health, physical functioning, bodily pain, emotional functioning, mental health, vitality, and social functioning. Each dimension is scored on a scale from 0–100, with higher scores indicating better quality of life.

##### 2.6.2.2 Laboratory tests

Included measurements of potassium, sodium, chloride, total protein, albumin, hemoglobin, and white blood cell count.

### 2.7 Evaluation and data collection

#### 2.7.1 Bowel function indicators

Collected at admission (T1), discharge (T2), and 1 month after discharge (T3).

#### 2.7.2 Quality of life indicators

Collected at admission (T1) and discharge (T2).

#### 2.7.3 Laboratory tests

Collected at admission (T1) and discharge (T2) from the hospital data platform.

### 2.8 Quality control

During the research design phase, a thorough review of national and international literature was conducted, and consultations with orthopedic experts helped formulate study design criteria to ensure scientific rigor. All team members received uniform training to assess the quality of the underlying literature and ensure reliable results. Experts were selected based on strict criteria, and clinical pre-testing was conducted to identify and address any potential issues.

During implementation, study subjects were selected according to the criteria, and the study protocol was strictly followed. Regular team meetings were held to address any issues arising during the intervention, and data collection was carried out following standardized guidelines. Patient information was accurately recorded to avoid errors, and any omissions were promptly addressed.

In the data analysis phase, all data were cross-checked by two individuals and entered into Excel. Outliers were identified and corrected as needed, and data analysis was performed using appropriate statistical methods.2.7 Ethical and informed consent.

### 2.9 Ethical considerations and informed consent

The study was designed to avoid any potential harm to patients, and all interventions were safe. Patients or their families were informed about the study and provided informed consent before participation. All collected data were used solely for research purposes and were not disclosed. The study was approved by the Biomedical Research Ethics Committee of the Affiliated Hospital of Zunyi Medical University (Approval No. LLY-2022-036).

Voluntary principle: patients or their families were informed of the content of this study prior to implementation in order to obtain consent and sign an informed consent form; Principle of non-harm: this study resolutely eliminates intentional and responsible harm, and all interventional items will not cause potential danger or harm to patients; Principle of confidentiality: all information collected in this study was only used for research purposes, and will never be disseminated; Ethical review: this study has been approved by the Affiliated Hospital of Zunyi Medical University Biomedical Research Ethics Committee, ethical review approval: LLY-2022-036.

### 2.10 Statistical analysis

Data analysis was performed using appropriate statistical methods. Data were analyzed using SPSS 29.0, with a significance level set at *p* < 0.05. Missing values were imputed based on the type of variable, using the mean for numerical variables and the mode for categorical variables. Descriptive statistics were used for count or rank data, and comparisons between groups were made using the chi-square test. Normally distributed measures were expressed as means ( $\bar{x}$ ± *s*), with *t*-tests used for between-group comparisons and analysis of variance (ANOVA) for within-group comparisons. Repeated-measures ANOVA was used to analyze data collected from patients at different time points. Furthermore, all tests were conducted with assumptions for each method carefully checked (e.g., normality and homogeneity of variance for *t*-tests and ANOVA). Adjustments were made as necessary (e.g., Welch’s ANOVA for unequal variances).

## 3 Results

### 3.1 Comparison of general data between the two groups

A total of 96 patients with neurogenic bowel dysfunction (NBD) after spinal cord injury (SCI) were included in this study, with 48 in the control group and 48 in the experimental group. Among them, in the control group, there were 17 females (35.42%) and 31 males (64.58%), with a mean age of 55.35 ± 12.593 years. The causes of injury were as follows: 27 cases (56.25%) of fall injuries, 6 cases (12.50%) of car accidents, 8 cases (16.67%) of falls from height, 1 case (2.08%) of crush injury, and 6 cases (12.50%) classified as other injuries. Regarding ASIA classification, there were 21 cases (43.75%) of grade A, 6 cases (12.50%) of grade B, 12 cases (25.0%) of grade C, and 9 cases (18.75%) of grade D. The average number of days of hospitalization was 16.79 ± 6.144 days.

In the experimental group, there were 20 females (41.67%) and 28 males (58.33%), with a mean age of 55.15 ± 12.848 years. The causes of injury were: 18 cases (37.50%) of fall injuries, 5 cases (10.42%) of car accidents, 15 cases (31.25%) of falls from height, 4 cases (8.33%) of crush injuries, and 6 cases (12.50%) classified as other injuries. For ASIA classification, there were 17 cases (35.42%) of grade A, 12 cases (25.0%) of grade B, 10 cases (20.83%) of grade C, and 8 cases (16.67%) of grade D. The average number of days of hospitalization was 15.71 ± 5.853 days. The results showed no statistically significant differences in the general information between the two groups (*p* > 0.05), indicating that the baseline information was comparable, as shown in Table 1.

**Table 1:** Comparison of general data between the two groups.

Project	Control group (*n* = 48)	Experimental group (*n* = 48)	*χ^2^/t*	*p*
Sex				
Woman	17 (35.42)	20 (41.67)	0.396	0.529
Man	31 (64.58)	28 (58.33)		
Age (year)	55.35 ± 12.59	57.15 ± 12.85	−0.690	0.492
Degree of education				
Primary school and below	31 (64.58)	33 (68.75)	0.596	0.742
Junior middle school	15 (31.25)	12 (25.00)		
High school and above	2 (4.17)	3 (6.25)		
Marital status				
Unmarried	2 (4.17)	4 (8.33)	0.178	0.673
Married	46 (95.83)	44 (91.67)		
Economic income				
Not have	31 (64.58)	20 (41.67)	5.750	0.056
<3,000	4 (8.34)	10 (20.83)		
≥3,000	13 (27.08)	18 (37.50)		
The type of residents				
Rural area	43 (89.58)	42 (87.50)	0.103	0.749
City	5 (10.42)	6 (12.50)		
Cause of injury				
Fall and hurt oneself	27 (56.25)	18 (37.50)	5.821	0.213
Car accident injury	6 (12.50)	5 (10.42)		
Fall from high	8 (16.67)	15 (31.25)		
Be injured by a crashing object	1 (2.08)	4 (8.33)		
Other	6 (12.50)	6 (12.50)		
Damage plane				
Neck	38 (79.16)	35 (72.92)	0.548	0.761
Chest	5 (10.42)	6 (12.50)		
Loin	5 (10.42)	7 (14.58)		
ASIA classify				
A	21 (43.75)	17 (35.42)	3.241	0.356
B	6 (12.50)	13 (27.08)		
C	12 (25.0)	10 (20.83)		
D	9 (18.75)	8 (16.67)		
Day of hospitalization (days)	16.79 ± 6.14	15.71 ± 5.85	0.885	0.379

### 3.2 Comparison of bowel function indicators in the two groups

#### 3.2.1 Comparison of bowel function counts between the two groups

As shown in Table 2, the difference in bowel function between the two groups of patients at the time of admission was not statistically significant (*p* > 0.05) and was comparable; the difference in abdominal distension and constipation at the time of discharge was statistically significant (*p* < 0.05). The difference in constipation between the two groups in the first month after discharge was statistically significant (*p* < 0.05) and the difference in abdominal distension and fecal incontinence was not statistically significant (*p* > 0.05).

**Table 2:** Comparison of bowel function indicators between the two groups (*n* = 96).

Project	Abdominal distension	Astriction	Incontinence of feces
On admission			
Control group	32 (66.67)	39 (81.25)	6 (12.50)
Experimental group	34 (70.83)	37 (77.08)	10 (20.83)
*χ^2^*	0.194	0.253	1.200
*P*	0.660	0.615	0.273
When discharged from hospital			
Control group	26 (54.17)	31 (64.58)	6 (12.50)
Experimental group	16 (33.3)	10 (20.83)	2 (4.17)
*χ^2^*	4.233	18.774	2.182
*P*	0.040	<0.001	0.140
She was discharged for 1 month			
Control group	33 (68.75)	35 (72.92)	4 (8.33)
Experimental group	27 (56.25)	22 (45.83)	3 (6.25)
*χ^2^*	1.600	7.298	0.154
*P*	0.206	0.007	0.695

#### 3.2.2 Comparison of bowel function measurements between the two groups

**Figure 1:**
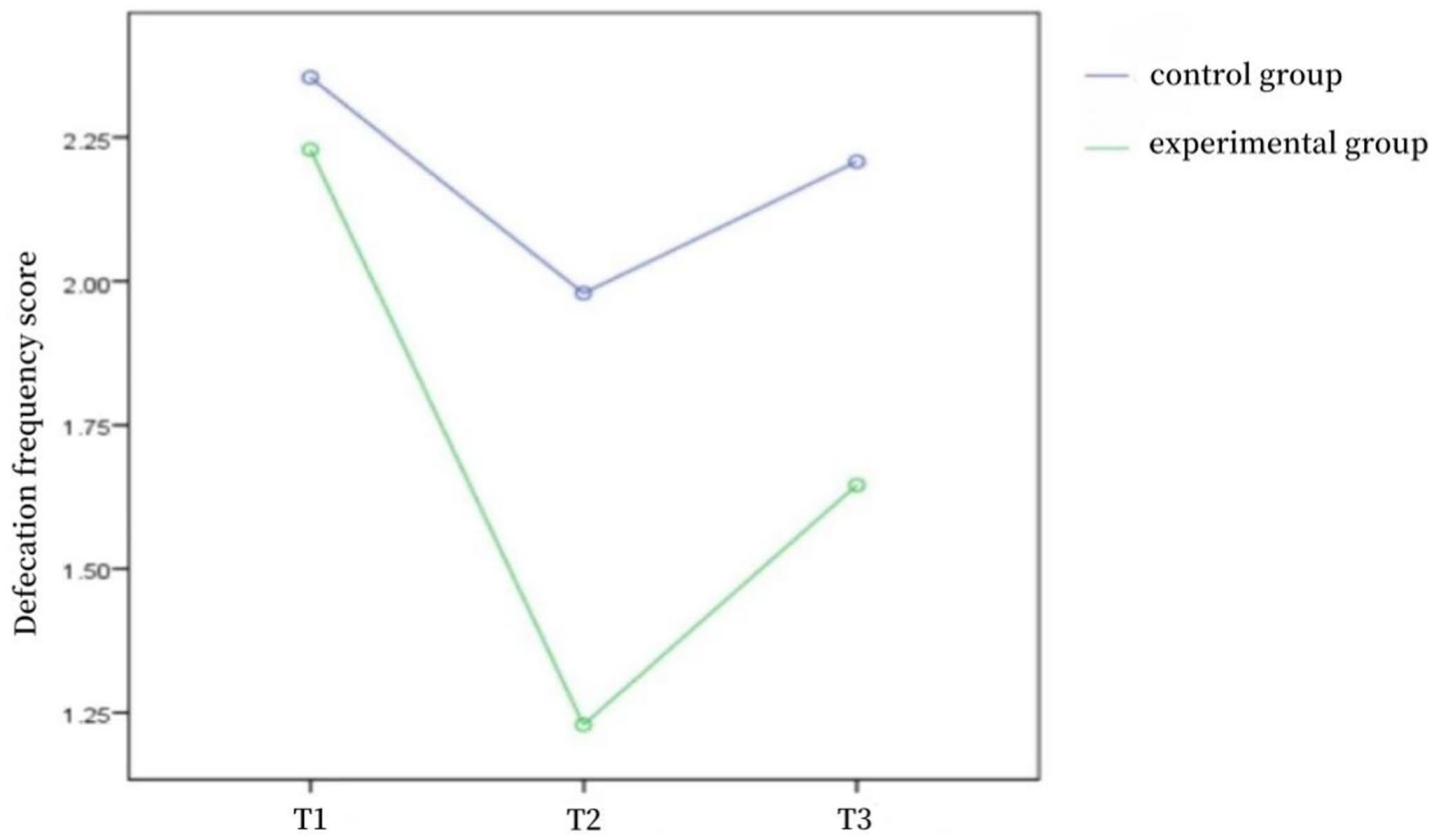
Defecation frequency score trend chart of two groups at different time points.

**Figure 2:**
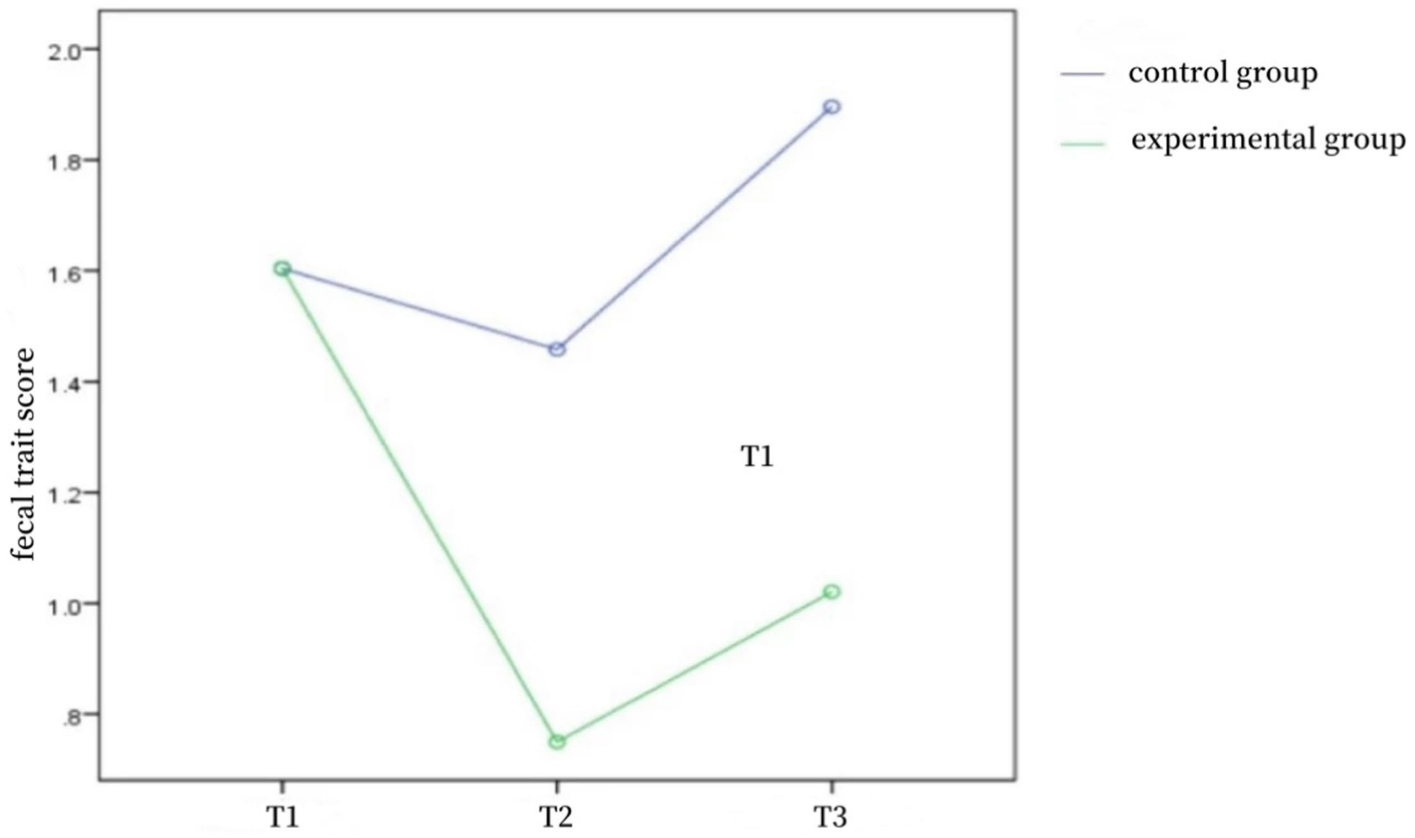
Fecal trait score trend chart of two groups at different time points.

**Figure 3:**
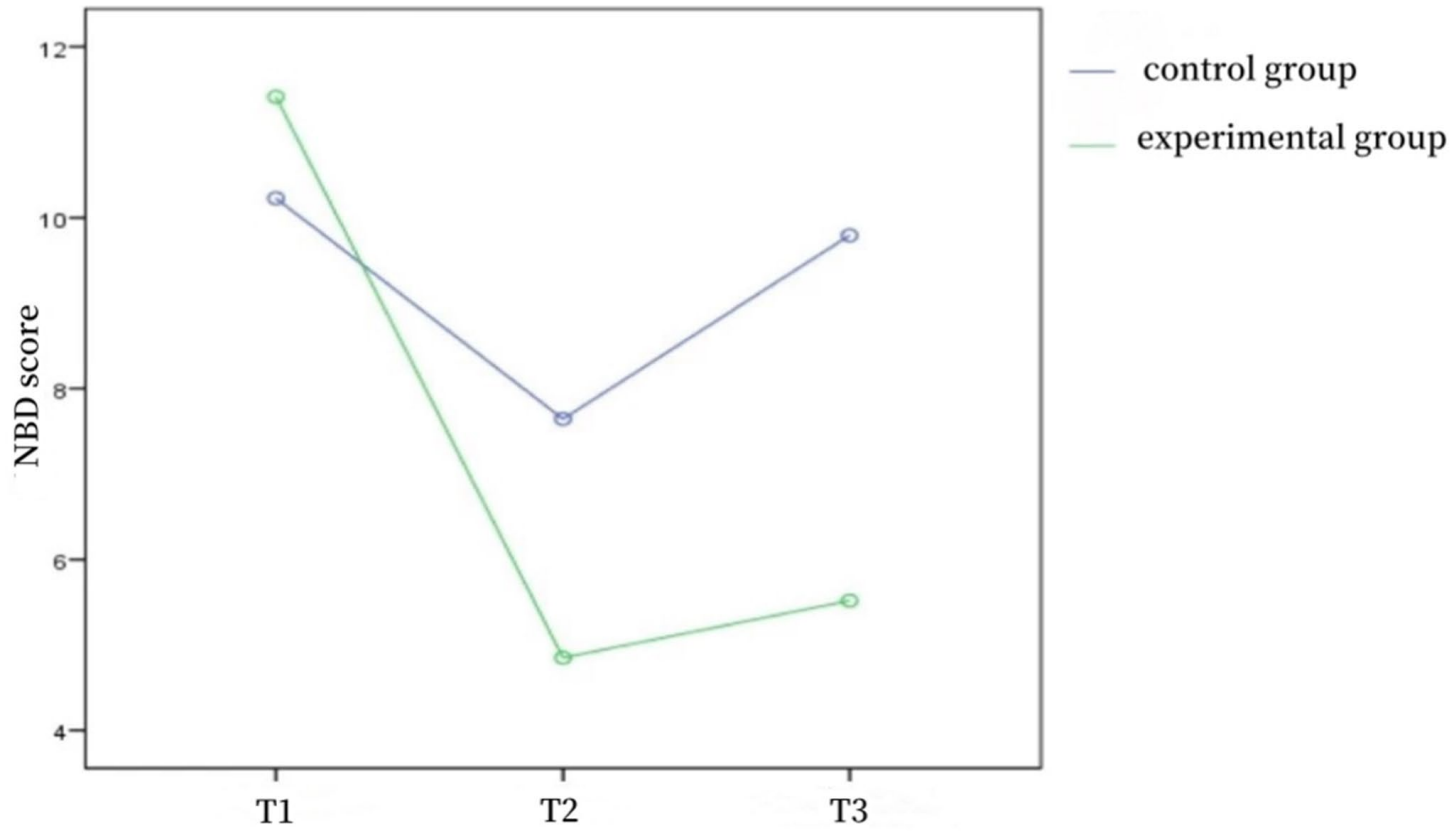
NBD score trend graph of two groups at different time points.

As shown in Table 3, the differences in defecation frequency scores, fecal character scores, and NBD scores between the two groups of patients at the time of admission were not statistically significant (*p* > 0.05) and were comparable; the differences in defecation frequency scores, fecal character scores, and NBD scores between the two groups at the time of discharge and 1 month after discharge were statistically significant (*p* < 0.05). Repeated-measures ANOVA was used to compare the defecation frequency scores, fecal character scores, and NBD scores of the two groups of patients before and after the intervention, and the results showed that the defecation frequency scores (*F* = 45.851, *p* < 0.001), fecal character scores (*F* = 11.263, *p* < 0.001), and NBD scores (*F* = 101.309, *p* < 0.001) of the control group and the experimental group were affected by the time factors, with a time effect, i.e., without considering the intervention factors, the frequency of defecation, fecal traits, and NBD scores of patients in the two groups changed over time; the frequency of defecation scores (*F* = 22.651, *p* < 0.001), fecal traits scores (*F* = 9.277, *p* < 0.001), and NBD scores (*F* = 4.693, *p* < 0.05) of the control group and the experimental group were affected by the intervention factors, with a group effect, i.e., without considering the time factor, the defecation frequency score, fecal character score, and NBD score of the two groups differed according to the intervention modality; in the control group and the experimental group, the defecation frequency score (*F* = 9.780, *p* < 0.001), the fecal character score (*F* = 10.593, *p* < 0.001), and the NBD score (*F* = 40.274, *p* < 0.001) time factor and intervention factors had an interaction effect, indicating that the effect of intervention factors on patients’ defecation frequency score, fecal trait score, and NBD score differed with the trend of time factor. The trends of defecation frequency scores, fecal character scores, and NBD scores at different time points in the two groups of patients are shown in Figures 1–3.

**Table 3:** Comparison between groups of defecation frequency scores, fecal trait scores, and NBD scores at different time points in the two groups (*n* = 96).

Group	On admission	When discharged from hospital	She was discharged for 1 month	*F* time	*F* divide into groups	*F* each other
Defecation frequency score						
Control group	2.35 ± 0.57	1.98 ± 0.70	2.21 ± 0.65	45.851^*^	22.651^*^	9.780^*^
Experimental group	2.23 ± 0.56	1.23 ± 0.63	1.65 ± 0.76			
*t*	1.094	5.533	3.901			
*P*	0.277	<0.001	<0.001			
Score of fecal traits						
Control group	1.6 ± 1.20	1.46 ± 1.18	0.90 ± 0.93	11.263^**^	9.277^**^	10.593^**^
Experimental group	1.6 ± 1.11	0.75 ± 0.64	7.58 ± 4.23			
*t*	0.000	3.651	4.605			
*P*	1.000	<0.001	<0.001			
NBD grade						
Control group	10.23 ± 5.22	7.65 ± 4.29	9.79 ± 5.21	110.902^**^	4.693^*^	40.274^**^
Experimental group	11.42 ± 5.53	4.85 ± 3.94	5.52 ± 4.22			
*t*	−1.082	3.322	4.414			
*P*	0.282	<0.001	<0.001			

^*^Representation *p* < 0.05, ^**^representation *p* < 0.001.

#### 3.2.3 Within-group comparison of bowel function indicators between the two groups

As shown in Table 4, the differences in bloating, constipation, fecal incontinence, and fecal character scores of the patients in the control group were not statistically significant (*p* > 0.05), and the differences in defecation frequency scores and NBD scores were statistically significant (*p* < 0.05); the differences in bloating, constipation, fecal incontinence, defecation frequency scores, fecal character scores, and NBD scores of the patients in the experimental group were statistically significant (*p* < 0.05).

**Table 4:** Comparison of bowel function indicators in the two groups (*n* = 96).

Group	Abdominal distension	Astriction	Incontinence of faeces	Defecation frequency score	Score of fecal traits	NBD grade
Control group						
On admission	32 (66.67)	39 (81.25)	6 (12.50)	2.35 ± 0.57	1.6 ± 1.20	10.23 ± 5.22
When discharged from hospital	26 (54.17)	31 (64.58)	6 (12.50)	1.98 ± 0.70^a^	1.46 ± 1.18	7.65 ± 4.29^a^
She was discharged for 1 month	33 (68.75)	35 (72.92)	4 (8.33)	2.21 ± 0.65	1.90 ± 0.93	9.79 ± 5.21^a^
*χ^2^/F*	2.568	3.376	0.563	4.179	1.932	3.781
*P*	0.277	0.185	0.755	0.017	0.149	0.025
Experimental group						
On admission	34 (70.83)	37 (77.08)	10 (20.83)	2.23 ± 0.56	1.6 ± 1.11	11.42 ± 5.53
When discharged from hospital	16 (33.33)^a^	10 (20.83)^a^	2 (4.17)^a^	1.23 ± 0.63^a^	0.75 ± 0.64^a^	4.85 ± 3.94^a^
She was discharged for 1 month	27 (56.25)	22 (45.83)^a^	3 (6.25)^a^	1.65 ± 0.76^ab^	1.02 ± 0.93^a^	5.52 ± 4.22^ab^
*χ^2^/F*	13.789	30.533	8.484	28.491	10.979	29.414
*P*	<0.001	<0.001	0.014	<0.001	<0.001	<0.001

A represents *p* < 0.05 for comparison with admission and b represents *p* < 0.05 for comparison with discharge.

### 3.3 Two groups, quality of life, index comparison

#### 3.3.1 Comparison of patient QoL between the two groups

As shown in Table 5, the difference in the quality of life between the two groups of patients at the time of admission was not statistically significant (*p* > 0.05) and was comparable; the difference in the quality of life between the two groups of patients at the time of discharge was statistically significant (*p* < 0.05).

**Table 5:** Comparison of quality of life between the two groups.

Project	General health	Physiological function	Physiological function	Somatic pain	Emotional function	Mental health	Vigor	Social function
On admission								
Control group	11.46 ± 12.59	7.81 ± 14.73	15.63 ± 32.87	34.38 ± 22.85	69.79 ± 42.20	34.38 ± 16.4	34.58 ± 15.29	23.75 ± 15.25
Experimental group	9.90 ± 12.36	9.38 ± 14.24	10.42 ± 20.52	38.54 ± 24.71	54.17 ± 45.93	40.42 ± 18.10	32.50 ± 12.80	26.67 ± 14.49
*t*	0.614	−0.528	0.931	−0.858	1.736	−1.710	0.724	−0.961
*P*	0.541	0.598	0.355	0.393	0.086	0.091	0.471	0.339
When discharged from hospital								
Control group	17.71 ± 11.48	10.42 ± 17.74	16.67 ± 33.16	65.10 ± 16.09	77.08 ± 39.89	46.88 ± 14.46	0.0035 ± 13.37	25.83 ± 14.27
Experimental group	40.63 ± 15.15	57.29 ± 33.41	71.88 ± 41.14	89.58 ± 12.46	92.71 ± 17.83	68.75 ± 18.86	66.25 ± 21.89	55.83 ± 21.02
*t*	−8.353	−8.586	−7.240	−8.333	−2.477	−6.376	−8.440	−8.181
*P*	<0.001	<0.001	<0.001	<0.001	0.015	<0.001	<0.001	<0.001

#### 3.3.2 Within-group comparison of quality of life in the two groups

As shown in Table 6, the differences between the patients in the control group were statistically significant in the dimensions of general health, somatic pain, and psychological health (*p* < 0.001), and the differences in the dimensions of physiological functioning, physical functioning, emotional functioning, vitality, and social functioning were not statistically significant (*p* > 0.05); and the differences in the quality of life of the patients in the experimental group in all dimensions were statistically significant (*p* < 0.001).

**Table 6:** Within-group comparison of patients in the two groups.

Group	General health	Physiological function	Physiological function	Somatic pain	Emotional function	Mental health	Vigor	Social function
Control group								
On admission	11.46 ± 12.59	7.81 ± 14.73	15.63 ± 32.87	34.38 ± 22.85	69.79 ± 42.20	34.38 ± 16.49	34.58 ± 15.29	23.75 ± 15.25
When discharged from hospital	17.71 ± 11.48	10.42 ± 17.74	16.67 ± 33.16	65.10 ± 16.10	77.08 ± 39.89	46.88 ± 14.46	35.00 ± 13.37	25.83 ± 14.27
*t*	−3.958	−1.401	−1.000	−11.800	−1.550	−6.440	−0.275	−1.044
*P*	<0.001	0.168	0.322	<0.001	0.128	<0.001	0.785	0.302
Experimental group								
On admission	9.90 ± 12.36	9.38 ± 14.24	10.42 ± 20.52	38.54 ± 24.71	54.16 ± 45.93	40.42 ± 18.10	32.50 ± 12.80	26.67 ± 14.49
When discharged from hospital	40.63 ± 15.15	57.29 ± 33.41	71.88 ± 41.14	89.58 ± 12.46	92.70 ± 17.83	68.75 ± 18.86	66.25 ± 21.89	55.83 ± 21.02
*t*	−15.343	−11.593	−10.940	−13.726	−6.632	−13.688	−15.063	−13.606
*P*	<0.001	<0.001	<0.001	<0.001	<0.001	<0.001	<0.001	<0.001

### 3.4 Comparison of laboratory test results between the two patient groups

#### 3.4.1 Comparison of laboratory results between two groups

As shown in Table 7, the difference in laboratory tests between the two groups of patients at the time of admission was not statistically significant (*p* > 0.05) and was comparable; the difference in laboratory tests of albumin and hemoglobin between the two groups of patients at the time of discharge was statistically significant (*p* < 0.001), and the difference in the results of potassium, sodium, chlorine, total protein, and leukocyte counts was not statistically significant (*p* > 0.05).

**Table 7:** Comparison of laboratory findings between the two groups.

Project	Potassium	Sodium	Chlorine	Total protein	Albumin	Hemoglobin	Leucocyte count
On admission							
Control group	3.99 ± 0.52	136.54 ± 4.17	105.33 ± 13.21	57.07 ± 8.80	37.64 ± 6.89	122.77 ± 19.11	10.63 ± 3.90
Experimental group	3.98 ± 0.46	137.56 ± 6.39	106.48 ± 6.25	58.68 ± 8.53	36.26 ± 5.16	118.69 ± 18.71	12.07 ± 4.08
*t*	0.063	−0.927	−0.543	−0.913	1.110	1.058	−1.771
*P*	0.950	0.356	0.588	0.364	0.270	0.293	0.08
When discharged from hospital							
Control group	4.16 ± 0.47	136.85 ± 4.90	102.65 ± 4.71	57.07 ± 5.98	32.99 ± 3.72	0.00110 ± 16.07	10.01 ± 3.57
Experimental group	4.08 ± 0.40	137.71 ± 2.84	102.88 ± 3.39	57.93 ± 4.02	0.0041 ± 6.30	130.13 ± 16.08	9.05 ± 2.17
*t*	0.936	−1.045	−0.274	−0.828	−7.586	−6.134	1.600
*P*	0.352	0.298	0.785	0.410	<0.001	<0.001	0.113

#### 3.4.2 Within-group comparison of laboratory test results between the two patient groups

As shown in Table 8, the differences in laboratory tests of albumin and hemoglobin in patients of the control group were statistically significant (*p* < 0.001), and the differences in potassium, sodium, chloride, total protein, and leukocyte counts were not statistically significant (*p* > 0.05); and the differences in chloride, albumin, hemoglobin, and leukocyte counts of patients of the experimental group were statistically significant (*p* < 0.001), and the differences in potassium, sodium, and total protein were not statistically significant (*p* > 0.05).

**Table 8:** Within-group comparison of laboratory findings between the two groups.

Project	Potassium	Sodium	Chlorine	Total protein	Albumin	Hemoglobin	Leucocyte count
Control group							
On admission	3.99 ± 0.52	136.54 ± 4.17	105.33 ± 13.21	57.07 ± 8.80	37.64 ± 6.90	122.77 ± 19.11	10.63 ± 3.90
When discharged from hospital	4.16 ± 0.47	136.85 ± 4.90	102.65 ± 4.71	57.07 ± 5.98	32.99 ± 3.72	0.00110 ± 16.07	10.01 ± 3.57
*t*	−1.985	−0.354	1.327	0.000	3.842	4.966	0.928
*P*	0.053	0.725	0.191	1.000	<0.001	<0.001	0.358
Experimental group							
On admission	3.98 ± 0.46	137.56 ± 6.39	106.48 ± 6.25	58.68 ± 8.53	36.26 ± 5.16	118.69 ± 18.71	12.07 ± 4.08
When discharged from hospital	4.08 ± 0.40	137.71 ± 2.84	102.88 ± 3.39	57.93 ± 4.02	0.0041 ± 6.30	130.13 ± 16.08	9.05 ± 2.17
*t*	−1.306	−0.209	3.568	0.585	−4.364	−5.124	5.276
*P*	0.198	0.836	<0.001	0.561	<0.001	<0.001	<0.001

## 4 Discussion

NBD following SCI can lead to significant neurological and functional impairments, resulting in symptoms such as bloating, constipation, and fecal incontinence. In addition, patients will also have reduced physical activity and independent defecation ability, resulting in varying degrees of difficulty in defecation or accidental defecation. Although the bowel intervention measures used in clinical practice have achieved certain curative effects, they do not fully meet the needs of patients, and lack of scientific and normative.

This study set up a multidisciplinary team to implement the bowel management program based on evidence-based nursing, expert meetings and pre-experiments, which is scientific and scientific. This program can dynamically assess the bowel function status of patients, so as to take timely and accurate targeted care. In this study, 68.75% of the 96 patients experienced abdominal distension, 79.17% had constipation, and 16.67% suffered from fecal incontinence at the time of admission. These findings align with previous research by Wei Xiaomei et al. (23), which reported similar incidences of these symptoms. The results showed that the intervention significantly reduced bloating and constipation at discharge (*p* < 0.05). However, the difference in constipation persisted one month post-discharge, which may be attributed to a decline in patient adherence after leaving the hospital. The intervention positively influenced defecation frequency, stool consistency, and NBD scores over time, though no significant improvement in fecal incontinence was observed, possibly due to the small sample size of patients with this condition. Within-group analysis revealed that in the control group, only defecation frequency and NBD scores showed significant improvement (*p* < 0.05), It shows that the existing clinical intervention measures are not effective and there are some shortcomings. In contrast, the experimental group demonstrated significant improvements in bowel function both at discharge and one month later (*p* < 0.05). These findings suggest that bowel management protocols implemented by the multidisciplinary team effectively enhances bowel function and mitigates disease progression in NBD patients.

NBD profoundly impacts the quality of life in SCI patients, second only to limb function loss and more severely than bladder or sexual dysfunction. It hampers social interaction and work, leading to anxiety and depression (28). At admission, both patient groups reported low quality of life, particularly in general health, emotional functioning, and mental health. This may be related to the severity of their conditions and the financial burden of care. The study found that the bowel management protocol significantly improved various dimensions of quality of life (*p* < 0.05), consistent with previous research. This improvement indicates that the protocol not only enhances bowel function but also facilitates social and work reintegration (15). However, the study only assessed quality of life up to the time of discharge, necessitating further investigation into its long-term effects.

Laboratory tests serve as biochemical markers of the nutritional status of NBD patients post-SCI. Patients with NBD are prone to malnutrition, leading to decreased levels of total protein, albumin, and hemoglobin, which can exacerbate their condition and hinder recovery (29, 30). The study found significant differences in albumin and hemoglobin levels between the two groups at discharge (*p* < 0.05), although no significant differences were observed in potassium, sodium, chloride, total protein, or white blood cell counts (*p* > 0.05). This may be due to malabsorption and high metabolic demands (31, 32). Within-group analysis showed that the experimental group experienced significant improvements in chlorine, albumin, hemoglobin, and white blood cell counts (*p* < 0.05), indicating that the bowel management protocol positively influenced these laboratory markers, though its effects on potassium, sodium, and total protein require further exploration.

## 5 Conclusion

In this study, demonstrated the clinical feasibility and effectiveness of a standardized bowel management protocol for NBD patients following SCI. The protocol significantly improved bowel function, reduced symptoms such as bloating, constipation, and fecal incontinence, and enhanced quality of life. Although the program is only used for a short time due to time constraints, the results suggest that this protocol could be a valuable addition to clinical practice, warranting further research to explore its long-term impact.

## Data Availability

The raw data supporting the conclusions of this article will be made available by the authors, without undue reservation.
