# Supplementary material for: Assessing the feasibility of a bowel management program for patients with neurogenic bowel function after spinal cord injury
Source: Front Med (Lausanne). 2025 Feb 5;12:1499184. doi: 10.3389/fmed.2025.1499184 (PMC11835857; doi:10.3389/fmed.2025.1499184)
Supplement: Supplementary file 1 [file Table_1.DOCX]

**Bowel management programm for patients with neurogenic bowel dysfunction after SCI**

| **First-level entry** | **Secondary entry** | **Level 3 entries** |
| --- | --- | --- |
| I-1 Bowel Assessment | II-1 Medical History Assessment | Timing of III-1 assessment: assessment within 24h of admission and before discharge. Reassessment using the Basic Data Set for Bowel Function should be done when the patient's disease worsens with changes in treatment (including diet and medications), blood in the stool, changes in stool properties and bowel movements longer than 1h. |
|  |  | III-2 Assessment content: the nurse in charge takes the patient's general information and history, including age, gender, mental status, diet, sleep, exercise, current bowel problems, bowel habits and pre-injury bowel function and defecation (frequency, time, colour, volume and stool character etc.), use of bowel intervention medications, and use of bowel interventions. |
|  | II-2 Severity Assessment | III-3 Timing of assessment: provided that the patient's vital signs are stable, it is recommended that the assessment be carried out within 24h of admission, 1 week after the intervention, and before discharge. |
|  |  | III-4 Assessment Content |
|  |  | III-4-1 The responsible physician uses a checklist to assess the severity of the spinal cord injury, the plane of damage, and the level of injury. III-4-1 The responsible physician uses the checklist to assess the severity of the spinal cord injury, the plane of impairment, and the level of impairment. |
|  |  | III-4-2 The charge nurse assesses the patient's current bowel dysfunction score using the International Spinal Cord Injury Bowel Function Basis Dataset and determines the severity of the bowel dysfunction based on the level of the score (4 scales: very mild 0-6, mild 7-9, moderate 10-13 and severe 14). |
|  | II-3 Assessment of Bowel Function | III-5 Abdominal Assessment |
|  |  | III-5-1 Timing of abdominal assessment: daily assessment during the patient's hospitalisation until the disappearance of gastrointestinal symptoms as well as pre-discharge assessment. |
|  |  | Ⅲ-5-2 Abdominal assessment content: visual examination, palpation, percussion, and auscultation: to find out whether the patient has flatulence, faecal impaction, abdominal pain, abdominal mass, and bowel peristalsis. |
|  |  | III-6 Bowel sounds assessment |
|  |  | III-6-1 The charge nurse assessed the patient's bowel sounds daily using a stethoscope to learn about the patient's bowel movements and to determine whether the patient was experiencing paralytic bowel obstruction. Assessment results: normal bowel sounds, decreased bowel sounds, and hyperactive bowel sounds. The assessment was stopped when the patient's paralytic bowel obstruction disappeared and the bowel sounds returned to normal. |
|  |  | III-7 Assessment of abdominal distension |
|  |  | III-7-1 The responsible nurse assesses the patient's abdominal distension daily and records the severity of the distension. Measurement of abdominal circumference: After the patient woke up in the morning and was instructed to take a flat lying position, a measuring tape was used to circle the abdomen with a flat umbilicus. Measurement criteria: mild abdominal distension increases abdominal circumference <10%, moderate abdominal distension increases abdominal circumference 10%-20%, The abdominal circumference of severe abdominal distension increased by > 20%, and the abdominal circumference of patients without abdominal distension remained unchanged. (Note: Normal male abdominal circumference ≤85cm, female abdominal circumference ≤80cm). |
|  | II-4 Other Supporting Assessments | III-8 Timing of assessment: the use of ancillary tests is determined by the responsible physician based on the patient's disease combination. |
|  |  | III-9 Content of assessment |
|  |  | III-9-1 magnetic resonance imaging: to show the degree of spinal cord integrity and specific pathological changes, and to clarify the site of spinal cord nerve injury as well as the severity of the injury. |
|  |  | III-9-2 Stool routine: to screen for bowel bacterial, viral and parasitic infections, white blood cells, red blood cells, bacterial sensitivity test and faecal occult blood in patients with spinal cord injury, which is used to identify diseases related to bowel dysfunction in patients with spinal cord injury. |
|  |  | III-9-3 Abdominal CT: to assess whether the patient's small intestine, colon and pelvic structures appear abnormal, which can be used to exclude other lesions that may cause gastrointestinal symptoms or constipation in patients with spinal cord injury. |
|  |  | III-9-4 Abdominal X-ray: assess patients with neurogenic bowel dysfunction for the presence of bowel distension, bowel sounds, organomegaly, fecal incontinence, site of faecal impaction and degree of loading. |
|  |  | III-9-5 Endoscopy: to observe the patient's intestines for structural changes and, if necessary, to take biopsies for testing for diagnosis or exclusion of colonic abnormalities and associated cancerous lesions. |
|  |  | III-9-6 Barium enema: observe the emptying time of barium in the large intestine, examine various occupying lesions in the large intestine, and clarify whether there is colorectal cancer and other diseases. |
| Ⅰ-2 Bowel Intervention | II-5 Bloating Intervention | III-10 Dietary Intervention |
|  |  | III-10-1 Target and timing of dietary intervention: patients with bowel dysfunction who have been assessed for mild, moderate or severe bloating. |
|  |  | III-10-2 Content of dietary intervention |
|  |  | III-10-2-1 Encourage patients with abdominal distension to eat foods that facilitate gas evacuation, such as meat, poultry, seafood, and other high proteins that do not contain carbohydrates per se, as well as fruits such as bananas and oranges. |
|  |  | III-10-2-2 Avoid flatulent foods such as milk and beans if possible, and limit fruits such as apples, pears, watermelon, and other fermentable carbohydrates. |
|  |  | III-10-2-3 It is recommended that enteral nutrition is preferred and gastrointestinal decompression is used in patients who cannot eat by mouth. |
|  |  | III-11 Abdominal massage |
|  |  | III-11-1 Abdominal massage targets and timing: patients with bowel dysfunction who have been assessed as having mild or moderate abdominal distension, usually after 30min after meals, 2-3 times a day, 15-20min each time. |
|  |  | III-11-2 Contents of abdominal massage |
|  |  | III-11-2-1 The patient was placed in the supine position, the knees were bent, and the responsible nurse applied the palm-sized fish posture from right to left |
|  |  | Rising colon, transverse colon, descending colon, sigmoid colon sequential massage abdomen, when massage to the left lower abdomen, increase pressure to the abdomen (to feel pain is appropriate). |
|  |  | III-11-2-2 For patients with severe abdominal distension, abdominal massage combined with medication can be used. Operation procedure: after abdominal massage, put 500g of mannite into a special bag, close it and apply it flatly on the patient's abdomen and fix it with abdominal band, the tightness should be appropriate for the patient to feel not tight and the mannite bag does not move when moving. Precautions: the nurse in charge of checking the abdominal skin once every 4h, 24h to replace 1, replace the mannitrides before a good abdominal skin care, the patient's abdomen and back skin with warm water to clean, conducive to the absorption of drugs. |
|  |  | Ⅲ-12 Anal tube excretion |
|  |  | Ⅲ-12-1 Anal tube excretion object and timing: patients who are assessed to have severe abdominal distension and cannot be relieved by abdominal massage, use anal tube excretion to reduce abdominal distension. |
|  |  | III-12-2 Contents of anal tube excretion |
|  |  | Ⅲ-12-2-1 responsible nurse to assist the patient to take the left lateral position, lubricate the front end of the anal tube gently inserted from the anus into the rectum 15-20cm when fixed in the buttocks with adhesive tape, the other end of the anal tube connected to the drain tube inserted into the water and keep it open, in addition to observing the drain tube with or without gas bubbles, when the exhaust is not smooth, then we need to assist the patient to change the position, massage the abdomen, so as to make the gas in the anal tube to be discharged out of the body, the anal tube is retained for at least 20min, pull the tube out of the body. The anal tube should be kept for at least 20min, and the perianal area should be wiped clean after removing the tube. |
|  |  | III-13 Gastrointestinal decompression |
|  |  | III-13-1 Gastrointestinal decompression Objects and timing: patients who have been assessed as having severe abdominal distension, use gastrointestinal decompression to reduce the pressure in the gastrointestinal tract. |
|  |  | III-13-2 Contents of gastrointestinal decompression: a method of inserting a gastric tube through the oral or nasal cavity, connecting a disposable gastrointestinal decompressor, and causing the gastric contents to be drawn out of the patient's body under the action of negative pressure and the principle of siphonage. |
| Ⅰ-2 Bowel Intervention | II-6 Constipation Intervention | III-14 Dietary Intervention |
|  |  | III-14-1 Who and when to intervene with diet: patients assessed as constipated by the Rome III diagnostic criteria or patients assessed as Type I and Type II using the Bristol Stool Trait Score. |
|  |  | III-14-2 Content of dietary intervention |
|  |  | III-14-2-1 Ensure that patients eat a regular, balanced diet every day, urge patients to eat more fruits and vegetables, and ensure that their daily fibre intake is 15g-40g. |
|  |  | Ⅲ-14-2-2 Encourage patients to drink ≥2000ml of water per day, about 4 bottles of mineral water. |
|  |  | III-14-2-3 Urge patients to eat a high-protein, high-energy diet, supplemented with dietary agents such as vitamin B12, vitamin D, and vitamin E, e.g., pig liver and vegetables. |
|  |  | III-14-2-4 Abstain from foods such as spicy and stimulating foods, and limit the intake of coffee, tea, and diuretics to avoid aggravating constipation or causing diarrhoea. |
|  |  | III-15 Behavioural Interventions |
|  |  | III-15-1 Behavioural intervention target and timing: patients assessed as constipated by Rome III diagnostic criteria. |
|  |  | III-15-2 Behavioural intervention content |
|  |  | III-15-2-1 Patients are advised to develop good defecation habits, preferably at a fixed time of the day and when the gastrocolic reflexes are at their strongest, usually 20-30 min after meals. The specific time of defecation should also be combined with the patient's personal life habits. |
|  |  | III-15-2-2 Under the premise that the condition permits, it is recommended that patients adopt their own daily habit of defecation, such as toilet, potty chair and other ways can better promote the patient's defecation. |
|  |  | III-15-2-3 Encourage patients to get out of bed as soon as their condition permits. For patients who cannot move out of bed, it is recommended to perform basic turning movements in bed to promote defecation. For paralysed patients, early guidance should be given to use the unparalysed limbs to do active functional exercises in bed, to do anal and abdominal exercises, and to strengthen the abdominal and pelvic floor muscles to promote bowel peristalsis, which can prevent constipation or promote defecation. |
|  |  | III-16 Abdominal massage is the same as bloating intervention. |
|  |  | III-17 Pharmacological intervention |
|  |  | Ⅲ-17-1 Pharmacological intervention target and timing: for patients with constipation for whom the above dietary, behavioural and abdominal massage interventions are ineffective, oral laxatives, gastrointestinal stimulants, rectal suppositories, enemas, etc., can be given as prescribed by the doctor; the timing should be 1 time per day or every other day |
|  |  | III-17-2 Pharmacological Intervention Components |
|  |  | Ⅲ-17-2-1 Oral laxatives: volumetric laxatives are preferred for patients assessed to be mildly constipated, and large amounts of water should also be consumed during the medication period to avoid colonic obstruction due to insufficient water intake; osmotic laxatives, such as lactulose and polyethylene glycol, are used for patients assessed to be chronically constipated or to have blockage of the stool. |
|  |  | III-17-2-2 Gastrointestinal dynamics drugs: commonly used drugs include mosapride, metoclopramide, erythromycin, etc. The use of this drug should follow a small dose and a short period of time to avoid causing nausea, vomiting and other adverse reactions. |
|  |  | Ⅲ-17-2-3 Rectal suppositories: rectal suppositories promote bowel peristalsis by stimulating the bowel wall, commonly used drugs are: kaiser, glycerol suppositories and so on. |
|  |  | III-17-2-4 Enemas: patients assessed for acute constipation or severe constipation may be induced to defecate by using small doses of enemas to stimulate the rectum and soften the faeces. |
|  |  | III-18 Valsalva manoeuvre and rectal stimulation |
|  |  | III-18-1 Valsalva action and rectal stimulation Intervention target: patients with bowel dysfunction (defecation reflexes present, lack of active control) assessed as having a spinal cord injury level of S2-4 or higher. |
|  |  | III-18-2 Valsalva movement and rectal stimulation intervention content |
|  |  | Ⅲ-18-2-1 Valsalva manoeuvre: the nurse in charge assists the patient to take a sitting or sloping lying position, lean forward with the abdomen relaxed, inhale deeply and then hold the breath for 10-12s, exerting force to transmit the abdominal pressure to the bladder, rectum and pelvic floor, increasing the abdominal pressure and causing the discharge of stool. Note Hypertension, cardiac arrhythmia or cardiac insufficiency, haemorrhoids patients are prohibited. |
|  |  | Ⅲ-18-2-2 Rectal stimulation: the responsible nurse assists the patient to take the left lateral lying position or sitting position, wear gloves, press the finger coated with lubricant longitudinally on the anal opening to relax the sphincter, then insert the finger gently into the rectum and massage and slowly stretch the anal canal along the rectal wall in a clockwise circular motion, to induce defecation reflexes, withdraw the finger after the end of stimulation, and wait for the patient to have a bowel movement. The duration of each rectal stimulation was 15s-1min, repeated once in 5-10min, and up to 3 times a day. If the stool still exists after repeating the operation for 3 times, the finger can be used to assist defecation. Precautions: before the operation, health care personnel should trim the nails gently to avoid damage to the rectal mucosa; during the operation, attention should be paid to protecting the patient's privacy; during the operation, keep communicating with the patient, and ask the patient to relax, so that the anal sphincter is in a state of relaxation, which is convenient for the feces to be discharged. |
|  |  | III-19 Finger-assisted defecation |
|  |  | III-19-1 Finger-assisted defecation Intervention object: patients with bowel dysfunction (no defecation reflex) assessed as having a spinal cord injury plane below S2-4. |
|  |  | III-19-2 Finger-assisted defecation intervention content |
|  |  | Ⅲ-19-2-1 Responsible nurses take finger-assisted defecation for patients with bowel dysfunction whose spinal cord injury level is below S2-4, assist the patient to take the left lateral recumbent position or sitting position, press the gloved and lubricated finger longitudinally at the anal opening to relax the sphincter muscle, then insert the finger into the anus gradually to mash the feces, and each time, pick out a small piece slowly until all the feces are taken out. Precautions are the same as for rectal stimulation. |
|  |  | III-19-2-2 Defecation environment: prepare a quiet and comfortable environment, evacuate other companions and visitors in the same ward if necessary, and dispose of excreta in time. Teach patients and family members to use the defecator correctly, and open the window in time to ventilate after defecation. |
|  | II-7 Fecal Incontinence Intervention | III-20 Intervention subjects: patients with fecal incontinence rated as type VI and type VII by the Bristol Stool Trait Score, and patients with lower motor neuron sexual injuries due to injuries below the level of the spinal cord cones. |
|  |  | III-21 Intervention content |
|  |  | III-21-1 Dietary intervention |
|  |  | III-21-1-1 The first step is to maintain water-electrolyte balance, eat meals on time, and replenish vitamins or electrolytes needed by the body in a timely manner to avoid hyponatraemia. |
|  |  | III-21-1-2 It is recommended to eat high-energy, high-protein foods to satisfy the gastrointestinal energy supply and supplement the body's catabolism. |
|  |  | III-21-1-3 Intake of excessive fibre, such as corn, mushrooms, kiwi and other foods that may cause diarrhoea in patients should be restricted. |
|  |  | III-21-1-4 Foods that may aggravate diarrhoea, such as chilli, hotpot and fritters, should be prohibited, such as spicy, stimulating and deep-fried foods. |
|  |  | III-21-2 Behavioural intervention: the responsible nurse instructs the patients with fecal incontinence to carry out pelvic floor muscle training, specific operation: the patient is instructed to independently and repeatedly perform the action of contracting the perineum and anal sphincter, and each time the state of contraction is maintained for 5-10s and then relaxes for 10s, and each group repeats 8-10 times, and the training is done in at least 4-5 groups per day. |
|  |  | III-21-3 Pharmacological intervention |
|  |  | III-21-3-1 Use medications for fecal incontinence such as montelukast, methylcobalamin, and bifidobacterium. |
|  |  | III-21-3-2 After each defecation of the patient, the nurse in charge should instruct the family to clean the perianal skin in a timely manner, check whether the patient's perianal skin is intact, and apply zinc oxide oil locally on the skin to protect the perianal skin mucosa. |
|  | II-8 Health Education | Ⅲ-22 Timing of education: after the patient's condition is stabilised on admission, before and after operation, and before discharge, respectively |
|  |  | Ⅲ-23 Educational content |
|  |  | III-23-1 The nurse in charge should introduce to the patient and/or family members the knowledge related to the disease of NBD after SCI, including the effects of spinal cord injury on the digestive tract and excretory function, the types of NBD and the causative factors. |
|  |  | III-23-2 Inform the patient and/or family of the specific contents of the bowel management programme, the purpose of each operation and the operation procedure, obtain the patient's consent and cooperation, and teach the patient or family to use techniques such as abdominal massage, measurement of abdominal circumference and finger-assisted defecation. |
|  |  | III-23-3 Teach patients and/or family members to use the Bristol Stool Trait Rating Scale, Rome III diagnostic criteria, and other methods of assessing constipation to facilitate self-monitoring after discharge. |
|  |  | III-23-4 Teach preventive and therapeutic measures for potential complications and provide psychological support and education. |
| Ⅰ-3Evaluation Indicators | II-9 Timing of Evaluations | III-24 Indicators of bowel function and quality of life at the time of admission, at the time of discharge, and 1 month after discharge. |
|  | II-10 Evaluation content | III-25 Laboratory test indicators at admission and at discharge. |
|  |  | III-26 Indicators of bowel function: including constipation, fecal incontinence, bowel frequency score, faecal character score and NBD score. |
|  |  | III-27 Laboratory tests: including Na+, K+, Cl^-^, haemoglobin, total protein, albumin and white blood cell count. |
|  |  | III-28 Quality of life: patients were assessed for improvement in quality of life using SF-12 scale. |
| Ⅰ-4 Discharge Follow-up | II-11 Discharge Guidance | III-29 Teach patients to self-assess and monitor the status of bowel function. |
|  |  | III-30 Instruct the patient in bowel management and the importance of discharge precautions. |
|  |  | III-31 Patients are taught about the review indicators and the importance of timely review. |
|  | II-12 Follow-up Management | III-32 Follow-up visits were regularly conducted once in 1-2 weeks after discharge for 1 month. |
|  |  | III-33 Follow-up visits included bowel functional symptoms, bowel conditions such as frequency of defecation, faecal character, duration of defecation, mode of defecation, use of medication, diet and water intake, constipation or fecal incontinence, and other bowel conditions. |
